# Sacrotuberous Ligament Healing following Surgical Division during Transgluteal Pudendal Nerve Decompression: A 3-Tesla MR Neurography Study

**DOI:** 10.1371/journal.pone.0165239

**Published:** 2016-11-09

**Authors:** Jan Fritz, Benjamin Fritz, A. Lee Dellon

**Affiliations:** 1 Russell H. Morgan Department of Radiology and Radiological Science, The Johns Hopkins University School of Medicine, 600 N Wolfe St., Baltimore, MD, 21287, United States of America; 2 Department of Radiology, Orthopedic University Hospital Balgrist, Forchstrasse 340, 8008, Zurich, Switzerland; 3 Department of Plastic and Reconstructive Surgery, The Johns Hopkins University School of Medicine, 600 N Wolfe St., Baltimore, MD, 21287, United States of America; Universita degli Studi di Roma La Sapienza Facolta di Medicina e Psicologia, ITALY

## Abstract

Pelvic pain due to chronic pudendal nerve (PN) compression, when treated surgically, is approached with a transgluteal division of the sacrotuberous ligament (STL). Controversy exists as to whether the STL heals spontaneously or requires grafting. Therefore, the aim of this study was to determine how surgically divided and unrepaired STL heal. A retrospective evaluation of 10 patients who had high spatial resolution 3-Tesla magnetic resonance imaging (3T MRI) exams of the pelvis was done using an IRB-approved protocol. Each patient was referred for residual pelvic pain after a transgluteal STL division for chronic pudendal nerve pain. Of the 10 patients, 8 had the STL divided and not repaired, while 2 had the STL divided and reconstructed with an allograft tendon. Of the 8 that were left unrepaired, 6 had bilateral surgery. Outcome variables included STL integrity and thickness. Normative data for the STL were obtained through a control group of 20 subjects. STL integrity and thickness were measured directly on 3 T MR Neurography images, by two independent Radiologists. The integrity and thickness of the post-surgical STL was evaluated 39 months (range, 9–55) after surgery. Comparison was made with the native contra-lateral STL in those who had unilateral STL division, and with normal, non-divided STL of subjects of the control group. The normal STL measured 3 mm (minimum and maximum of absolute STL thickness, 2–3 mm). All post-operative STL were found to be continuous regardless of the surgical technique used. Measured at level of Alcock’s canal in the same plane as the obturator internus tendon posterior to the ischium, the mean anteroposterior STL diameter was 5 mm (range, 4–5 mm) in the group of prior STL division without repair and 8 mm (range, 8–9 mm) in the group with the STL reconstructed with grafts (p<0.05). The group of healed STLs were significantly thicker than the normal STL (p<0.05). We conclude that a surgically divided STL will heal spontaneously and will be significantly thicker after healing.

## Introduction

For the most well-described peripheral nerve entrapment, the median nerve in the carpal tunnel, it has been demonstrated, with CT imaging that the transverse carpal ligament reforms after carpal tunnel release, but that the overall volume of the carpal tunnel is increased [1]. The most common operation utilized to decompress the pudendal nerve (PN) is a transgluteal surgical division of the sacrotuberous ligament (STL) [2–6]. In order to improve the outcome for neurolysis of the PN in the region of the STL, it is important to understand the pathophysiology and anatomic relationships of the STL to the PN and the effects of dividing the STL upon the PN morphology. These relationships have not been described utilizing 3-Tesla MR Neurography correlated with intra-operative views of the STL.

Following carpal tunnel release, symptoms of “pillar pain” have been recorded and attributed to small changes in the carpal bones [7–9], primarily on the radial side of the carpal canal. Following STL release for neurolysis of the PN, sacroiliac joint pain/dysfunction has been reported to occur [10, 11]. Attempts to reconstruct the transverse carpal ligament have been included in the neurolysis of the median nerve to prevent pillar pain [12, 13], and attempts have now been reported to reconstruct the STL following pudendal neurolysis [14, 15]. If post-operative imaging of the STL demonstrated that this ligament, like the transverse carpal ligament, also reformed, or “healed”, but that the PN demonstrated a more normal physiologic appearance post-operatively, then there would be evidence in support of not reconstructing the STL. Therefore, the aim of this study was to determine how surgically divided and unrepaired STL heal.

## Materials and Methods

Approval was obtained from the Johns Hopkins Medicine Institutional Review Boards (IRB00075143). The informed consent requirement was waived due to the retrospective nature of the study and minimal risk to the subjects. Our study was compliant with the Health Insurance Portability and Accountability Act of the United States of America, and with the Committee on Publication Ethics (COPE), and our study abides by its Code of Conduct, and aims to adhere to its Best Practice Guidelines.

A retrospective cohort was selected by researching our electronic health record system between the dates of July 2013 to December 2015. Search criteria included 3 Tesla MR Neurography (MRN) of the pelvis and history of pudendal nerve decompression surgery.

The search derived a cohort of 10 subjects (4 female, 6 male, mean age, 45 years; range, 22–62 years). MRN of the pelvis was obtained 39 months (range, 9–55) after surgery for various reasons. Of the 10 subjects, 8 subjects had undergone division of the STL without attempted reconstruction. Of these 8 subjects, 6 had the STL divided bilaterally and 2 had it divided unilaterally. The remaining 2 subjects underwent unilateral STL reconstruction with allograft tendon and the pudendal nerve wrapped with a collagen membrane, as described in their operative reports. In order to evaluate the STL of subjects that underwent bilateral STL surgery, twenty age-, bodyweight-, and MRN-protocol-matched subjects without history of surgery and without symptoms that were attributable to pudendal nerve pain were selected and added to the cohort. Indications of the control group to undergo MRN included neuropathy of the lateral femoral cutaneous (13/20, 65%), obturator (3/20, 15%) and anterior femoral cutaneous (4/20, 20%) nerves.

All subjects had undergone high spatial resolution MRN of the pelvis performed on a clinical wide-bore 3 Tesla MR imaging system (MAGNETOM Skyra, Siemens Healthcare, Erlangen, Germany). The protocol included three-dimensional intermediate-weighted and T2-weighted high-resolution turbo spin echo pulse sequences without and with fat suppression [16, 17].

All MRN studies were blinded and randomized with a computer algorithm. Two experienced observers evaluated the MRN studies independently. Outcome variables included the integrity of the STL (divided or continuous) as visualized on multiplanar MRN images and the anteroposterior thickness of the STL at level of Alcock’s canal in the same plane as the obturator internus tendon posterior to the ischium. Measurements were performed using the electronic caliper tool of picture archiving and communications system software (Ultravisual, Merge Healthcare, Chicago, IL).

Statistical analyses were performed using JMP Pro 11.1.1 software (SAS Institute, Cary, NC, USA). Continuous variables are given as the median with minimum and maximum in parentheses. Kappa statistics were used to assess the observer agreement of STL integrity and intra-class correlation coefficients were used to assess the observer agreement of the STL thickness measurements. Non-parametric testes were used to assess for group differences. p-values of 0.05 and less were considered statistically significant. STL integrity was assessed visually and the STL dimensions were measured directly on the 3 T MRI, by two independent Radiologists.

## Results

**Fig 1:**
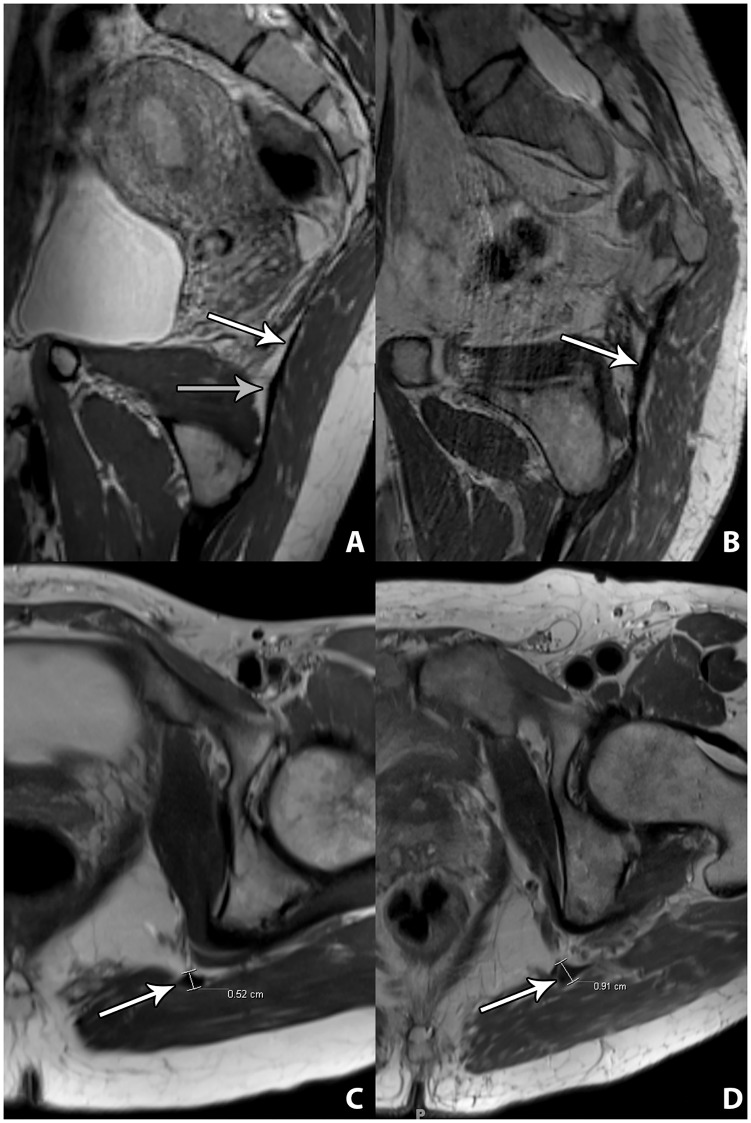
Three-dimensional 3-Tesla MR Neurography of the healed sacrotuberous ligament following complete division and grafting. A: Sagittal oblique multiplanar intermediate-weighted MR image demonstrates continuity of the healed sacrotuberous ligament (STL) (white arrow) after previous complete division, indicative of spontaneous healing. The gray arrow indicates the healed division site. B: Sagittal oblique multiplanar intermediate-weighted MR images demonstrate continuity of a STL allograft (white arrow). C: Two-dimensional 3-Tesla MR Neurography of the healed STL following complete division and grafting, at level of Alcock’s canal. Axial intermediate-weighted MR image demonstrates the anteroposterior dimensions of the healed STL (white arrow) after previous complete division. D: Axial intermediate-weighted MR image the anteroposterior dimensions of the STL allograft (white arrow).

The analysis included 14 STL following division only, and two STL that had been reconstructed with allograft. There were no observer disagreements in regards to the assessments of the STL integrity (kappa = 1.00). There was continuity of all post-operative STL, regardless of prior STL division only (Fig 1) or prior STL grafting (Fig 1). As there was very high inter-observer agreement of the measurements of the STL thickness (interclass correlation coefficient: 0.923), the measurements of the two observers were combined in order to simplify data presentation. The average anteroposterior diameter of previously divided STL was 5 (minimum and maximum of absolute STL thickness, 4–5) mm (Fig 1). The average anteroposterior diameter of STL allografts was 8 (8–9) mm (Fig 1). The average anteroposterior diameter of native STL was 3 (2–3) mm. The native STL was significantly thinner than the post-surgical STL (p<0.05). Healed divided STL were significantly thinner than STL allografts (p<0.05).

**Fig 2:**
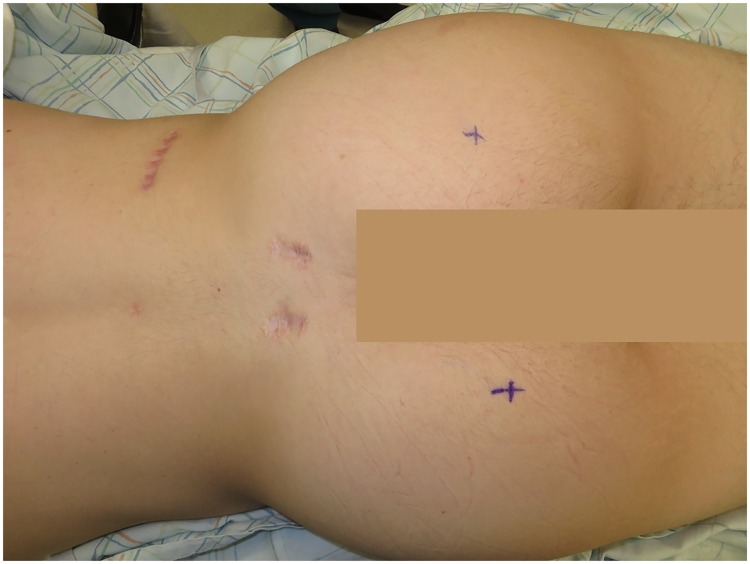
Pre-operative view. Patient prone. Note scars from previous spinal cord 17 stimulator surgery. The right and left asterisk is the site of the tender pudendal 18 nerve at the posterior border of the sacrotuberous ligament.

**Fig 3:**
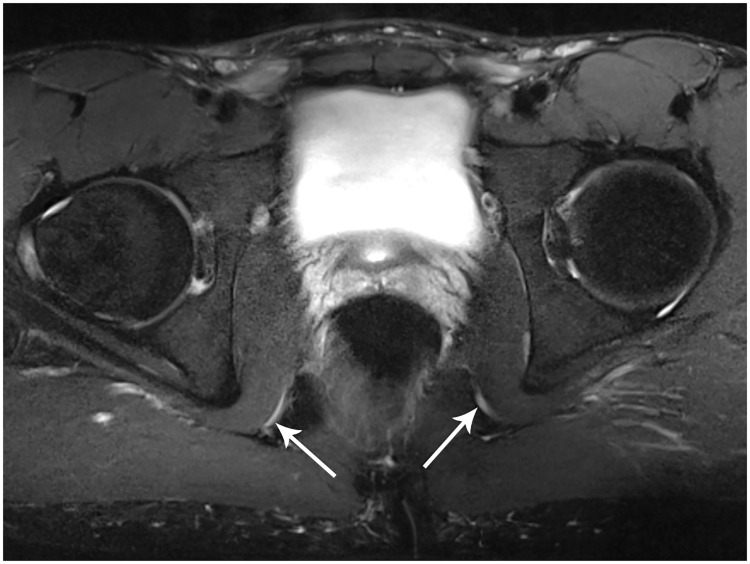
Pre-operative pudendal nerve 3T MR neurography. Axial high spatial resolution T2-weighted MR image with fat suppression demonstrate abnormally hyperintense and thickened pudendal nerves bilaterally (arrows).

**Fig 4:**
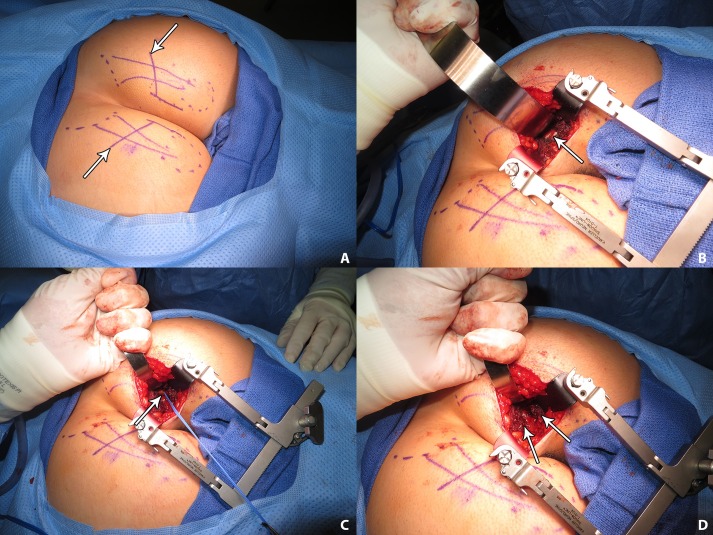
Intra-operative photographs of sacrotuberous ligament and pudendal nerve, right side, with patient in prone position. A: Bilateral markings to indicate the sacrotuberous ligament (STL) (vertical lines) connecting the sacrum (dashed lines) to the ischial tuberosity (dashed circles), with the proposed angled incision from the ischiorectal fossa (arrows), then crossing the STL. B: The intact STL (arrow) is noted between the gluteus maximus muscle fibers. C: A blue vessel loop is about the pudendal nerve (arrow) exiting the STL. D: The blackened, cauterized, edges of the STL (arrows) are separated about 1.5 to 2.0 cm after division of the ligament.

**Fig 5:**
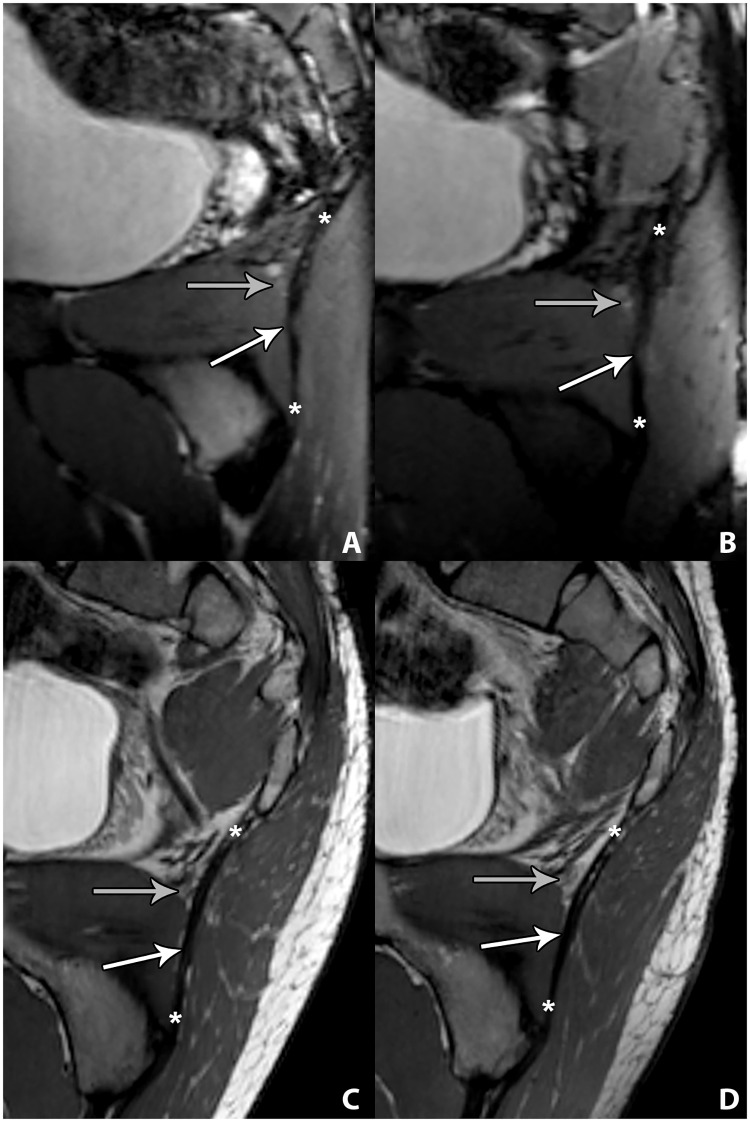
Post-operative pudendal nerve three-dimensional 3-Tesla MR Neurography of the sacrotuberous ligament 3 months (A) and 8 months (B) following sacrotuberous ligament division. A: Sagittal oblique multiplanar T2-weighted MR images with fat suppression demonstrate the continuous left sacrotuberous ligament (STL) (asterisks) with healing division site (white arrow). The gray arrow demonstrates the pudendal nerve subjacent to the STL. B: Sagittal oblique multiplanar T2-weighted MR images with fat suppression demonstrate the continuous right sacrotuberous ligament (STL) (asterisks) with healing division site (white arrow). The gray arrow demonstrates the pudendal nerve subjacent to the STL. C: Sagittal oblique multiplanar intermediate-weighted MR images demonstrate the continuous left STL (asterisks) with healed division site (white arrow). The gray arrow demonstrates the pudendal nerve subjacent to the STL. D: Sagittal oblique multiplanar intermediate-weighted MR images demonstrate the continuous left STL (asterisks) with healed division site (white arrow). The gray arrow demonstrates the pudendal nerve subjacent to the STL.

**Fig 6:**
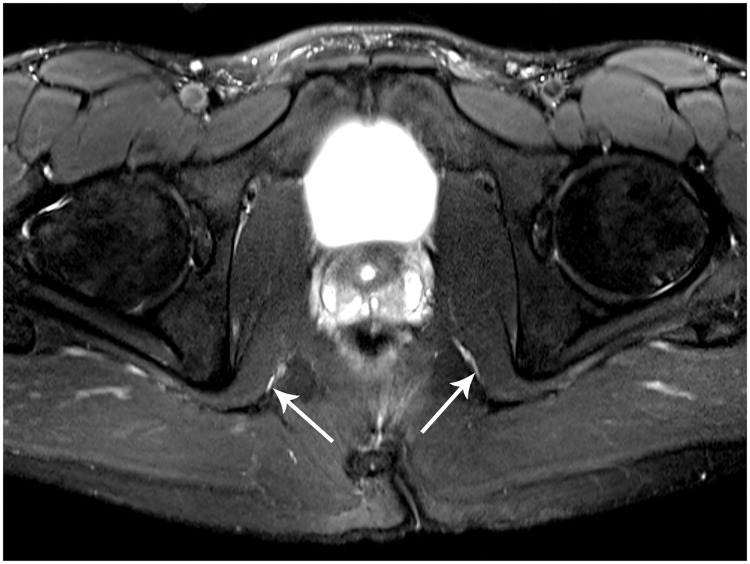
Post-operative pudendal nerve 3T MR neurography. Axial high spatial resolution T2-weighted MR image with fat suppression demonstrate near complete resolution of pudendal nerve engorgement (arrows) and decreased signal hyperintensity, consistent with interval improvement of symptoms.

One subject of our cohort, for which 3-Tesla MR Neurography exams of the pelvis were available prior to and after STL division, was selected as the index case as it documents the natural history of STL division with respect to healing or staying divided in a representative fashion: The Index Patient was a 23-year-old man when first seen in consultation and reported 22 months of worsening pelvic pain in the distribution of the pudendal nerve. The most likely cause had been an athletic injury, related to either wrestling, soccer, or baseball. He previously had surgery on both shoulders and one knee related to sports injuries, and these healed without problems. His symptoms included left-sided numbness in the penis, rectal pain, and perineal pain. He had already had treatment for chronic prostatitis by an Urologist, and had already had a sacral stimulator placed. His physical examination (Fig 2) demonstrated tenderness over the left exit of the Alcock canal, and a 3-Tesla MR Neurography demonstrated thickened and congested pudendal nerves bilaterally (Fig 3). After a pudendal nerve block, he had relief of symptoms. Therefore, he had an anterior approach to resect the perineal branch of the PN and implant the nerve into the obturator internus muscle, and a neurolysis of the dorsal branch of the pudendal nerve.^18^ Following this surgery, the symptoms in the penis and scrotum were relieved, but the rectal symptoms remained. These included worsening of rectal pain with defecation. He had an evaluation by the gastroenterologist specializing in manometrics, and these were normal. At this point he had a transgluteal division of the STL bilaterally (Fig 4). He has had three 3-Tesla MR Neurography exams 3 months and 8 months after STL division. After STL division, his rectal symptoms were relieved. The 3-month post-op MR Neurography demonstrated healing of the STL (Fig 5A), and the 8 month po-op MR Neurography documented a healed STL with the pudendal nerve beneath it without evidence of compression (Fig 5B) and improved MR Neurography appearance (Fig 6). At one year follow-up, he remained free of his rectal symptoms.

## Discussion

To the best of our knowledge, this study is the first to evaluate STL healing following division during transgluteal pudendal nerve decompression surgery. The results demonstrate that the STL, like the transverse carpal ligament, heals spontaneously after surgical division. This has implications for the surgical approach to transgluteal pudendal neurolysis. The implication is that the STL does not need to be reconstructed.

Much of the poor results attributed to transgluteal PN decompression is related to misdiagnosis of the symptoms being related to the pudendal nerve at the STL, the site of compression most commonly described in the surgical literature [2–6]. More recently, an anterior site, related to injury to the perineal and compression of the dorsal branch of the pudendal nerve, has been reported for pudendal nerve symptoms that do not include rectal pain [18]. Another possible source of failure of transgluteal pudendal decompression is the belief that the STL should be reconstructed due to its importance in pelvic stability [14, 15]. A review of the critical biomechanical studies that have been done relating the STL and the sacrospinous ligament to pelvic stability demonstrates that all of the four studies used cadavers, appropriate experimental methods, engineering principles, and showed that in the total of 35 pelvises the sequential division of the STL and sacrospinous ligament did *not* have any effect on pelvic stability, which was maintained by the much stronger and more important anterior and posterior sacroiliac ligaments [19–23]. Since the measurements of the two patients in the present study who had STL reconstruction demonstrated that their STL was 4 mm thicker than those healed STL that were not reconstructed, it is possible the STL reconstruction may reduce the available space for gliding of the PN.

A limitation of the present study is its small sample size, which mandated limited statistical comparison of these two groups.

## Conclusions

It is concluded that a surgically divided STL will heal spontaneously and will be significantly thicker after healing.
